# Intermediate Effects of Body Mass Index and C-Reactive Protein on the Serum Cotinine- Leukocyte Telomere Length Association

**DOI:** 10.3389/fnagi.2021.827465

**Published:** 2022-01-18

**Authors:** Xiao Gao, Yawei Kong, Shengxu Li, Shiqiu Dong, Xinyu Huang, Deyu Qi, Tao Zhang, Yinkun Yan, Wei Chen

**Affiliations:** ^1^The Second Clinical Medical College, Heilongjiang University of Chinese Medicine, Harbin, China; ^2^Center for Non-communicable Disease Management, Beijing Children’s Hospital, Capital Medical University, National Center for Children’s Health, Beijing, China; ^3^Children’s Minnesota Research Institute, Children’s Minnesota, Minneapolis, MN, United States; ^4^Department of Clinical Medicine, Heilongjiang Nursing College, Harbin, China; ^5^Department of Biostatistics, School of Public Health, Shandong University, Jinan, China; ^6^Department of Epidemiology, School of Public Health and Tropical Medicine, Tulane University, New Orleans, LA, United States

**Keywords:** cotinine, smoking, telomere length, body mass index, C-reactive protein, third variable model

## Abstract

We aimed to examine the association between serum cotinine and leukocyte telomere length (LTL) and the intermediate effects of body mass index (BMI) and C-reactive protein (CRP) on modulating the association. This study included 4,047 adults from the 1999 to 2002 National Health and Nutrition Examination Survey. In the combined sample, after adjusting for age, race, sex, physical activity, and alcohol use, the total effect of serum cotinine on LTL was significant (standardized regression coefficient, β = −0.049, *p* = 0.001) without BMI and CRP included in the model. With inclusion of BMI but without CRP in the model, the direct effect of cotinine on LTL in its absolute value increased to β = −0.053 (*p* < 0.001), and the suppression effect of BMI was estimated at 8.8%. With inclusion of CRP but without BMI in the model, the direct effect of cotinine on LTL in its absolute value decreased to β = −0.040 (*p* = 0.008), and the mediation effect of CRP was estimated at 16.9%. With inclusion of both BMI and CRP in the model, BMI and CRP still had significant suppression and mediation effects, respectively, on the cotinine-LTL association. These findings suggest that weight and inflammation have different roles in the inverse association between serum cotinine and LTL.

## Introduction

Telomeres are nucleotide repeats and a protein complex at the ends of chromosomes and shorten with each round of DNA replication (Blackburn, 2000). Shortened leukocyte telomere length (LTL) is hypothesized to be a biomarker for aging and age-related diseases, such as cardiovascular disease and cancer (von Zglinicki and Martin-Ruiz, 2005). Studies have demonstrated that cardiovascular risk factors such as sex, race, obesity, unhealthy behaviors, increased inflammation, and oxidative stress are associated with age-dependent LTL attrition (von Zglinicki, 2002; O’Donovan et al., 2011; Sanders and Newman, 2013; Rode et al., 2014; Starkweather et al., 2014; Shin et al., 2019).

Many studies have shown that tobacco smokers are more likely to have shorter telomeres (Sanders and Newman, 2013; Starkweather et al., 2014; Wulaningsih et al., 2016; Astuti et al., 2017; Khan et al., 2019). Smoking is known to continuously increase the cumulative burden of chronic inflammation and oxidative stress (van der Vaart et al., 2004; Madsen et al., 2007; Yanbaeva et al., 2007; Kianoush et al., 2017), leading to accelerated shortening of telomeres (von Zglinicki, 2002; O’Donovan et al., 2011; Rode et al., 2014; Shin et al., 2019). Despite the trend of shorter LTL with smoking found in the majority of previous studies, there are, however, considerable inconsistencies in the reported associations as summarized in a systematic review of 84 studies (Astuti et al., 2017). The role of a third variable, among various reasons, is a potential explanation for these discrepancies in the findings.

It has been well-known that cigarette smoking is inversely and positively associated with body weight and C-reactive protein (CRP), respectively (Molarius et al., 1997; van der Vaart et al., 2004; Madsen et al., 2007; Yanbaeva et al., 2007; Lv et al., 2015; Kianoush et al., 2017). Further, obesity and increased CRP are closely associated with LTL shortening (O’Donovan et al., 2011; Sanders and Newman, 2013; Müezzinler et al., 2014; Rode et al., 2014; Starkweather et al., 2014; Mundstock et al., 2015; Gielen et al., 2018; Shin et al., 2019; Gao et al., 2021). Clearly, body weight and CRP as third external variables are intermediate in the causal pathway from smoking to LTL and may play important roles in modulating the smoking-LTL relationship. The Bogalusa Heart Study has reported that body mass index (BMI) is a suppressor, not a mediator, on the smoking-LTL association (Yun et al., 2019). To date, data are very limited on the complex relationships between smoking, BMI, CRP, and LTL. There is a paucity of knowledge concerning the nature of the indirect effects through BMI and CRP on the smoking-LTL association.

This study aims to dissect direct effect of serum cotinine and its indirect effect through BMI and CRP on LTL to measure their suppression and mediation effects on the cotinine-LTL association in a quantitative manner in Black and White adults from the National Health and Nutrition Examination Surveys (NHANES), 1999–2002.

## Materials and Methods

### Study Population

NHANES aims to assess the health and nutritional status of non-institutionalized population in the United State. It is a continuous, multistage cross-sectional survey sponsored by the National Center for Health Statistics (NCHS). Details of the design, protocols, and methods of the surveys can be found at http://www.cdc.gov/nchs/nhanes.htm. Based on the 1999–2002 NHANES, we included in the current study 4,047 adults (3,114 Whites and 933 Blacks; 52.6% males; mean age = 50.3 years ranging from 20.0 to 84.9 years) with data available on serum cotinine, CRP, BMI, and LTL and with no missing data on age, sex, race, physical activity, alcohol use, and cigarette smoking. Mexican Americans (*n* = 1,404) were not included in the current analysis due to a significant difference in the total effect of serum cotinine on LTL compared to Whites. The associations between serum cotinine and LTL showed opposite directions, with the total effect of cotinine on LTL measured as standardized regression coefficients (SE) being −0.056 (0.017) in Whites and 0.025 (0.026) in Mexican Americans (*p* = 0.014 for the cotinine-race interaction). We chose to report the results in the total cohort by combing race groups in this study. Subgroups that have significant interaction between the predictor and group cannot be combined for analysis based on the statistical rules. Other races (*n* = 460) were not included because the sample size was small in multiple subgroups. All study protocols were reviewed and approved by the NCHS Institutional Review Board.

### Laboratory Measurements

The LTL assay was performed in the laboratory of Elizabeth Blackburn at the University of California, San Francisco. Details of the assay method for the current study are available elsewhere (Needham et al., 2015; Wulaningsih et al., 2016; Shivappa et al., 2017; Khan et al., 2019). In brief, quantitative polymerase chain reaction was used to measure LTL relative to standard reference DNA (T/S ratio). To convert the T/S ratio to absolute length (base pairs), the formula (3,274 + 2,413 × T/S ratio) was used. Serum cotinine was determined by an isotope dilution-high performance liquid chromatography/atmospheric pressure chemical ionization tandem mass spectrometry (ID HPLC-APCI MS/MS) (Benowitz et al., 2009). Serum CRP was measured at the University of Washington Medical Center Department of Laboratory Medicine with a Dade Behring Nephelometer II Analyzer system (Dade Behring Diagnostics) (Shivappa et al., 2017).

### Covariates

Validated questionnaires were used to collect data on demographics, alcohol drinking, physical activity, and cigarette smoking. Weight was measured in light clothing without shoes to the nearest 0.1 kg in a Brecknell Weight and Height Physician Scale; height was measured to the nearest 0.1 cm with a free-standing stadiometer. BMI was calculated as weight in kilograms divided by height in meters squared. Detailed descriptions of the methods are available at http://www.cdc.gov/nchs/nhanes.htm. Questions “Do you now smoke cigarettes?” and “how many cigarettes did you smoke per day during the past 30 days?” were asked at the time of the interview. Current smokers were defined as those who smoked at least 1 cigarette every day.

### Statistical Methods

Differences in continuous and categorical study variables were tested using analyses of covariance and chi-square test, respectively, between race and sex groups. Serum CRP and cotinine were log-transformed due to their skewed distributions. The associations of log-cotinine, log-CRP and BMI with LTL were examined in multivariable linear regression analysis models, adjusted for age, sex, race, physical activity, and alcohol drinking. Because second-hand smoking also contributes to serum cotinine levels, we performed sensitivity analyses with self-reported current cigarette smoking as a replacement for serum cotinine.

The general third variable model (MacKinnon et al., 2000) was constructed to examine the indirect effects through BMI and CRP on the association between serum cotinine and LTL, adjusted for covariates. Log-cotinine was the predictor variable (X), BMI and/or log-CRP the third variables (Z), and LTL the dependent variable (Y). Z stands for either a mediator or a suppressor. In general, there are four steps for the third variable model analysis: (1) showing that cotinine determines LTL (Y = c X) where c is total effect; (2) showing that cotinine affects BMI and/or CRP (Z = β_1_ X) where β_1_ is indirect effect 1; (3) showing that cotinine determines LTL controlling for BMI and/or CRP (Y = β_2_ Z + c’ X) where β_2_ is indirect effect 2, and c’ is direct effect. Overall indirect effect (β_Ind_) is calculated as β_1_ × β_2_; (4) determining mediation effect and suppression effect by comparing the magnitude (the absolute values) of the total effect (c) and direct effect (c’). In the case of |c| > |β_Ind_| in this study, a mediation effect is suggested if |c| > |c’|, and a suppression effect is suggested if |c| < |c’|. The mediation effect (%) is calculated as |β_Ind_|/|c| × 100%, and the suppression effect (%) as |β_Ind_|/|c’| × 100%. Significance of the overall indirect effect was tested using the method previously described (MacKinnon et al., 2002). Differences in the model parameters between race and sex groups were tested for significance in interaction regression models by including respective interaction terms.

## Results

### Participant Characteristics

The characteristics by race and sex are displayed in Table 1. Although log-transformed cotinine and CRP were used for subsequent analyses, their original values are presented in Table 1. The mean levels of continuous variables were compared between subgroups, adjusting for age (except age itself). All study variables showed significant sex and race differences except for sex differences in smoking and BMI in Whites.

**TABLE 1:** Descriptive data of study participants by race and sex.

	Whites		Blacks	
	Male (*n* = 1,630)	Female (*n* = 1,484)	Male (*n* = 500)	Female (*n* = 433)
Age (year)	52.8 (17.8)	49.2 (18.2)^a^	48.6 (16.0)	46.5 (16.1)^a,b^
Physical activity, *n* (%)		^a^		^a,b^
No	911 (55.9)	824 (55.5)	340 (68.0)	296 (68.4)
Moderate	468 (28.7)	507 (34.2)	79 (15.8)	89 (20.6)
Vigorous	251 (15.4)	153 (10.3)	81 (16.2)	48 (11.1)
Alcohol use, *n* (%)		^a^		^a,b^
Never	428 (26.3)	491 (33.1)	161 (32.2)	195 (45.0)
≤1 day/week	591 (36.3)	696 (46.9)	195 (39.0)	177 (40.9)
2–3 days/week	274 (16.8)	154 (10.4)	76 (15.2)	39 (9.0)
4–7 days/week	337 (20.7)	143 (9.6)	68 (13.6)	22 (5.1)
Current smoker, n (%)	416 (25.5)	364 (24.5)	191 (38.2)	123 (28.4)^a,b^
Serum cotinine (ng/mL)	76 (137)	48 (101)^a^	116 (164)	76 (135)^a,b^
Serum CRP (mg/dL)	0.35 (0.69)	0.51 (0.72)^a^	0.52 (1.19)	0.67 (0.99)^a,b^
BMI (kg/m^2^)	27.9 (5.1)	27.6 (6.7)	28.0 (6.2)	31.3 (8.0)^a,b^
LTL (bp)	5,700 (626)	5,796 (640)^a^	5,818 (639)	5,957 (673)^a,b^

*Means (standard deviation) are presented unless otherwise indicated. CRP, C-reactive protein; BMI, body mass index; LTL, leukocyte telomere length.*

*^a^p < 0.05 for sex difference with race groups.*

*^b^p < 0.05 for race difference.*

### Associations of Cotinine, Body Mass Index and C-Reactive Protein With Leukocyte Telomere Length

The associations between log-cotinine, BMI, log-CRP and LTL in 6 models are presented in Table 2 in terms of standardized regression coefficients (β), adjusted for covariates. Model 1 shows a significant effect of cotinine on LTL without BMI and CRP included in the model. When BMI was added in Model 2, effects of cotinine and BMI on LTL were significant, and the effect of cotinine increased in its absolute value compared to the value in Model 1. In Model 3, effects of cotinine and CRP on LTL were significant, and the effect of cotinine decreased in its absolute value compared to the value in Model 1. In Model 4 with both CRP and BMI included, effects of cotinine, CRP and BMI on LTL were significant. Models 5 and 6 show significant effects of cotinine on BMI and CRP. When self-reported smoking was used in separate models as a predictor to replace serum cotinine in the sensitivity analysis, the total effect of smoking on LTL was negative and significant. The effect sizes of CRP, BMI and covariates did not change substantially and had the same directions of the associations (Supplementary Table 1).

**TABLE 2:** Standardized regression coefficients of serum cotinine, BMI and CRP on LTL.

	Model 1 β (95% CI)	Model 2 β (95% CI)	Model 3 β (95% CI)	Model 4 β (95% CI)
Age	−0.399** (−0.429, −0.369)	−0.397** (−0.427, −0.367)	−0.384** (−0.414, −0.354)	−0.386** (−0.416, −0.356)
Black race	0.072** (0.042, 0.102)	0.079** (0.049, 0.109)	0.076** (0.046, 0.106)	0.080** (0.050, 0.110)
Female sex	0.051** (0.021, 0.081)	0.052** (0.022, 0.082)	0.064 ** (0.034, 0.094)	0.062** (0.032, 0.092)
Active PA	0.045** (0.017, 0.073)	0.043** (0.015, 0.071)	0.040** (0.012, 0.068)	0.040** (0.012, 0.068)
Alcohol use	0.045** (0.015, 0.075)	0.039** (0.009, 0.069)	0.040** (0.010, 0.070)	0.038* (0.008, 0.068)
Log-CRP	−	−	−0.075** (−0.105, −0.045)	−0.061** (−0.093, −0.029)
BMI	−	−0.059** (−0.089, −0.029)	−	−0.036* (−0.068, −0.004)
Log-cotinine	−0.049** (−0.079, −0.019)	−0.053** (−0.083, −0.023)	−0.040 ** (−0.070, −0.010)	−0.045** (−0.075, −0.015)

*β, standardized regression coefficients; CI, confidence interval; PA, physical activity; CRP, C-reactive protein; BMI, body mass index; LTL, leukocyte telomere length.*

*p < 0.05 and **p < 0.01 for βs being different from 0.

*Model 5: Effect of Log-cotinine on BMI: β −0.079 (−0.111, −0.047), p < 0.001.*

*Model 6: Effect of Log-cotinine on log-CRP: β = 0.109 (0.077, 0.141), p < 0.001.*

### Suppression Effect of Body Mass Index

**FIGURE 1:**
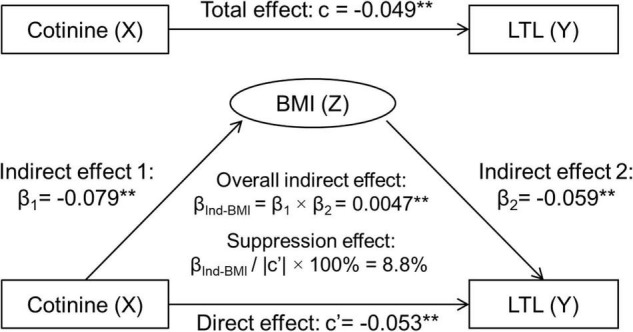
Third variable model of serum cotinine, BMI and LTL, adjusting for age, race, sex, physical activity, and alcohol drinking. β, c and c’ are standardized regression coefficients. c, total effect; c’, direct effect; β_1_, indirect effect 1; β_2_, indirect effect 2; β_Ind–BMI_, overall indirect effect; BMI, body mass index; LTL, leukocyte telomere length. **p < 0.01 for β, c, and c’ being different from 0. In the case of |c| > |β_Ind–BMI_|: Total effect (c) = Direct effect (c’) + Indirect effect (β_Ind–BMI_). −0.0486 = −0.0533 + 0.0047, |c| < |c’|, then β_Ind–BMI_ has a suppression effect.

Figure 1 depicts the results of third variable model analyses with inclusion of BMI, adjusted for covariates. The overall indirect effect (β_Ind–BMI_) through BMI on the cotinine-LTL association, the product of β_1_ and β_2_, was estimated at β_Ind–BMI_ = 0.0047, *p* = 0.002. The direct effect of BMI on LTL (c’ = −0.0533, *p* < 0.001) was greater than the total effect (c = −0.0486, *p* = 0.001) in terms of their absolute values. The suppression effect was estimated at 0.0047/|−0.0533| × 100% = 8.8%.

### Mediation Effect of C-Reactive Protein

**FIGURE 2:**
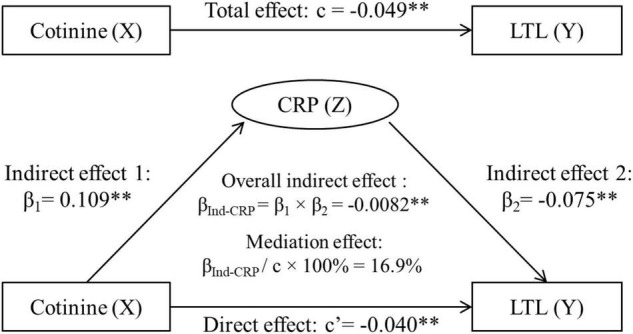
Third variable model of serum cotinine, CRP and LTL, adjusting for age, race, sex, physical activity, and alcohol drinking. β, c, and c’ are standardized regression coefficients. c, total effect; c’, direct effect; β_1_, indirect effect 1; β_2_, indirect effect 2; β_Ind–CRP_, overall indirect effect; CRP, C-reactive protein; LTL, leukocyte telomere length. ^**^p < 0.01 for β, c, and c’ being different from 0. In the case of |c| > |β_Ind–CRP_|: Total effect (c) = Direct effect (c’) + Indirect effect (β_Ind–CRP_). −0.0486 = −0.0404 + (−0.0082), |c| > |c’|, then β_Ind–CRP_ has a mediation effect.

Figure 2 displays the mediation model analyses with inclusion of CRP, adjusted for covariates. The overall indirect effect (β_Ind–CRP_) through CRP on the cotinine-LTL association was estimated at β_Ind–CRP_ = −0.0082, *p* < 0.001. The direct effect of cotinine on LTL (c’ = −0.0404, *p* = 0.008) was smaller than the total effect (c = −0.0486, *p* = 0.001) in terms of their absolute values. The mediation effect was estimated at |−0.0082|/|−0.0486| × 100% = 16.9%.

### Effects of Body Mass Index and C-Reactive Protein in One Model

**FIGURE 3:**
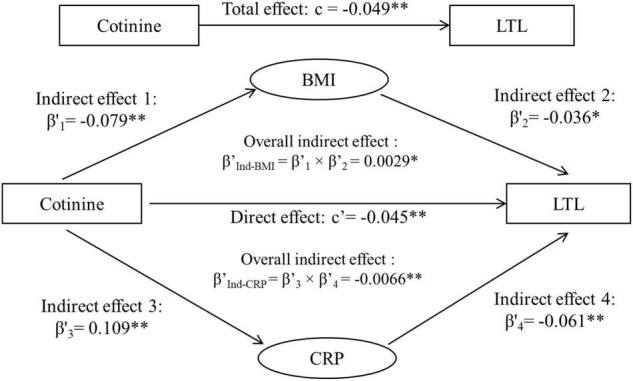
Third variable model of serum cotinine, BMI, CRP and LTL, adjusting for age, race, sex, physical activity, and alcohol drinking. β’, c, and c’ are standardized regression coefficients. c, total effect; c’, direct effect; β’s, indirect effect; β’_Ind_, overall indirect effect; BMI, body mass index; CRP, C-reactive protein; LTL, leukocyte telomere length. *p < 0.05 and ^**^p < 0.01 for β’, c, and c’ being different from 0. Total effect (c), Direct effect (c’) + Indirect effect (β’_Ind–BMI_) + Indirect effect (β’_Ind–CRP_). −0.0486 = −0.0448 + 0.00285 + (−0.0066).

Figure 3 shows the third variable model analyses with both BMI and CRP included, adjusted for covariates. The total effect (c) is the sum of three components of direct effect (c’), indirect effect through BMI (β’_Ind–BMI_) and indirect effect through CRP (β’_Ind–CRP_), that is, −0.0448 + 0.00285 + (−0.0066) = −0.0486. The overall indirect effects of cotinine through BMI (β’_Ind–BMI_) and through CRP (β’_Ind–CRP_), its direct effect (c’) and associations of cotinine with BMI and CRP were all significant.

### Effects by Race and Sex

In Table 3, the total, direct and indirect effects of cotinine on LTL did not differ significantly between race and sex groups except for indirect effect 1 between races and indirect effects 1 and 3 between sexes.

**TABLE 3:** Differences in effects of serum cotinine, BMI and Log-CRP on LTL between race and sex groups.

	Cotinine→LTL (c)^a^	Cotinine→BMI (β’_1_)	BMI→LTL (β’_2_)	Cotinine→CRP (β’_3_)	CRP→LTL (β’_4_)	Cotinine→LTL (c’)^b^
Whites (*n* = 3,114)	−0.056**	−0.064**	−0.036*	0.114**	−0.065**	−0.051**
Blacks (*n* = 933)	–0.021	−0.117**	–0.049	0.076*	–0.046	–0.024
P^d^	0.497	0.003	0.991	0.359	0.418	0.631
Males (*n* = 2,130)	−0.068**	−0.139**	–0.014	0.188**	−0.064**	−0.058**
Females (*n* = 1,917)	–0.031	–0.026	−0.066**	0.029	–0.045	–0.032
P^e^	0.570	<0.001	0.188	<0.001	0.255	0.758

*β’, c, and c’ are standardized regression coefficients. BMI, body mass index; CRP, C-reactive protein; LTL, leukocyte telomere length.*

*^a^BMI and CRP were not included in the models.*

*^b^BMI and CRP were included in the models.*

*^d^P-value for race difference.*

*^e^P-value for sex difference.*

*p < 0.05 and **p < 0.01 for β’, c, and c’ being different from 0.

## Discussion

In the current study, we examined in what way and to what extent BMI and CRP levels modulated the association between LTL and serum cotinine, an indicator of current exposure to tobacco smoke, in Black and White adults from the 1999 to 2002 NHANES. The central findings are that BMI and CRP had different roles in the association between cotinine and LTL by showing suppression and mediation effects, respectively. Furthermore, the direct effect of cotinine on LTL remained significant after the indirect effects through BMI and CRP were removed. An important question raised in the present study deserves further discussion, that is, whether statistical adjustment for BMI is required to remove its suppression effect on the smoking/cotinine-LTL association. LTL is highly variable at birth and afterward due to influence of prenatal and postnatal factors (Slagboom et al., 1994; Okuda et al., 2002; von Zglinicki, 2002; Akkad et al., 2006; O’Donovan et al., 2011; Sanders and Newman, 2013; Rode et al., 2014; Starkweather et al., 2014; Shin et al., 2019). The findings in this study suggest that smoking, BMI and CRP and their complex relationships play a crucial role in age-dependent LTL attrition after birth.

Upon a significant association between a predictor variable and an outcome, special attention has to be paid to the influence of a third external variable on the relationship. Confounding, suppressing and mediating effects of a third variable on the predictor-outcome association were discussed in a general third variable model (MacKinnon et al., 2000). The confounding effect is a conceptual definition. The mediation and suppression effects can be distinguished by comparing the magnitude of the relationships between the predictor and outcome before and after the third variable is introduced in the models. We found that BMI had a suppression effect on the cotinine-LTL association, which replicated the results from the Bogalusa Heart Study that the strength of the smoking-LTL association was largely dependent on adjustment for BMI (Yun et al., 2019). Although there is distinction between a confounder and a suppressor in epidemiological concepts, they share statistical similarities. The suppression effect is analogous to a negative confounding in statistical modeling (MacKinnon et al., 2000). The findings of this study have more implications in the statistical aspect by suggesting that inclusion of BMI in the model is critical for demonstrating the direct effect of smoking on LTL with removal of the intervening variable effect.

In examining a mediational hypothesis, the relationship between a predictor variable and an outcome variable is decomposed into two causal paths as shown in Figure 2. A mediation effect implies that the indirect effect through the third variable is part of the total effect (MacKinnon et al., 2000). In the current study, CRP, by definition (Weinberg, 1993), is a mediator, not a confounder, for the cotinine-LTL association because it is intermediate in the causal path between cotinine and LTL. Cigarette smoking with high serum cotinine levels causes an increase in CRP (van der Vaart et al., 2004; Madsen et al., 2007; Yanbaeva et al., 2007; Kianoush et al., 2017) which then leads to LTL attrition (von Zglinicki, 2002; O’Donovan et al., 2011; Sanders and Newman, 2013; Rode et al., 2014; Starkweather et al., 2014; Shin et al., 2019; Gao et al., 2021). The new findings on the mediation effect of CRP in this study have more implications in the biological context by suggesting that smoking-induced increase in inflammation is one of the links between smoking and LTL. The significant direct effect after removing the effect of CRP indicates that smoking affects LTL attrition also through other biological mechanistic paths.

In the current study, we chose to present results of the association and third variable model analyses using serum cotinine instead of self-reported smoking as a predictor. Serum cotinine, a major metabolite of nicotine, can reflect current tobacco smoking and exposure to environmental tobacco smoke (Hukkanen et al., 2005; Gan et al., 2015) and does not have the bias of self-reported smoking history in the questionnaire data. Notably, the prevalence of self-reported smoking based on the 1999–2002 NHANES questionnaire data varied to some extent among previous studies (Wulaningsih et al., 2016; Khan et al., 2019) and the current study. The quality of the self-reported data, different definitions of smokers and the use of different variables were responsible for these discrepancies. Serum cotinine is more objective and reliable than the questionnaire data and was used to confirm self-reported current smoking and exposure to second-hand smoke (Wulaningsih et al., 2016; Khan et al., 2019). However, the disadvantage of using serum cotinine is that it does not reflect either active or passive exposure to nicotine in the past. In our sensitivity analyses by using self-reported smoking as a predictor to replace serum cotinine, the results did not change markedly.

Blacks are known to have longer LTL than other race groups (Hunt et al., 2008; Chen et al., 2009; Sanders and Newman, 2013). In the current study, we observed significant differences in LTL, serum cotinine, BMI and CRP between Whites and Blacks; however, the total and direct effects of serum cotinine on LTL did not differ significantly between race groups. With respect to sex differences, males than females showed stronger paths of from cotinine to BMI and CRP. To date, there have been no data available for reference and comparison regarding race-specific suppression and mediation analyses of BMI and CRP on the cotinine-LTL association.

The main strengths of the current study included a large sample, multiple race groups and availability of serum cotinine data. There were also several limitations. The NHANES data used in this study were from a cross-sectional design. The data with one-time assessment were not as reliable as longitudinal data. Further studies are needed to replicate the findings from our study. We speculate that multiple inflammation markers may be involved in mediating the cotinine-LTL relationship; however, we only analyzed CRP in our mediation analyses because the data on other inflammatory markers were incomplete or not available in the 1999–2002 NHANES. Further research is needed on the potential roles that other inflammation markers play in mediating the cotinine -LTL relationship. In addition, serum cotinine was used as the predictor in the association and third variable model analyses. The use of serum cotinine has both advantage and disadvantage as mentioned above. Lastly, Mexican Americans, and other races were not included in the current analysis due to significant race difference in the total effects of serum cotinine on LTL and small sample size, respectively. It should be with caution to generalize the study findings to other race/ethnicity populations.

In summary, these data provide strong and fresh evidence that elevated BMI and inflammation play different roles in linking tobacco smoking and accelerated biological aging. In the case that there are no external third variables included in the model, the total effect of serum cotinine on LTL is a mixed parameter and can be partitioned into three components as shown in Figure 3: (1) the suppression effect of BMI, (2) the mediation effect through CRP, and (3) the direct effect of serum cotinine on LTL. The findings from this study emphasize the statistical importance of removing any potential suppression effect from the total effect to elucidate the nature of the predictor-outcome relationship. The clinical significance is to improve the understanding of the biological mechanisms linking tobacco smoking and accelerated aging through weight status and inflammatory conditions and to underscore the importance of targeting smoking and its intermediate factors to delay the aging process and promote population health.

## Data Availability Statement

The original contributions presented in the study are included in the article/Supplementary Material, further inquiries can be directed to the corresponding author/s.

## Author Contributions

XG: conceptualization, methodology, formal analysis, writing – original draft, investigation, visualization, and writing – review. YK, SL, SD, XH, TZ, and DQ: investigation, writing – review, and editing. YY and WC: participated in supervision, conceptualization, editing, and funding acquisition. All authors read and approved the final manuscript.

## Conflict of Interest

The authors declare that the research was conducted in the absence of any commercial or financial relationships that could be construed as a potential conflict of interest.

## Publisher’s Note

All claims expressed in this article are solely those of the authors and do not necessarily represent those of their affiliated organizations, or those of the publisher, the editors and the reviewers. Any product that may be evaluated in this article, or claim that may be made by its manufacturer, is not guaranteed or endorsed by the publisher.

## Abbreviations

BMI: body mass index
CRP: C-reactive protein
LTL: leukocyte telomere length
NHANES: National Health and Nutrition Examination Survey.
