# Supplementary material for: Intermediate Effects of Body Mass Index and C-Reactive Protein on the Serum Cotinine- Leukocyte Telomere Length Association
Source: Front Aging Neurosci. 2022 Jan 18;13:827465. doi: 10.3389/fnagi.2021.827465 (PMC8806079; doi:10.3389/fnagi.2021.827465)
Supplement: Supplementary file 1 [file Table_1.DOCX]

|  | **Model 1**  β (95% CI) | **Model 2**  β (95% CI) | **Model 3**  β (95% CI) | **Model 4**  β (95% CI) | |
| --- | --- | --- | --- | --- | --- |
| Age | -0.394 **  (-0.424, -0.364) | -0.393 **  (-0.423, -0.363) | -0.379 **  (-0.409, -0.349) | | -0.381 **  (-0.411, -0.351) |
| Black race | 0.067 **  (0.037, 0.097) | 0.074 **  (0.044, 0.104) | 0.072 **  (0.044, 0.100) | | 0.075 **  (0.045, 0.105) |
| Female sex | 0.056 **  (0.026. 0.086) | 0.057 **  (0.027, 0.087) | 0.069 **  (0.039, 0.099) | | 0.067 **  (0.037, 0.097) |
| Active PA | 0.045 **  (0.017, 0.073) | 0.044 **  (0.016, 0.072) | 0.041 **  (0.013, 0.069) | | 0.041 **  (0.013, 0.069) |
| Alcohol use | 0.044 **  (0.014, 0.074) | 0.038 **  (0.008, 0.068) | 0.039 **  (0.009, 0.069) | | 0.037 *  (0.007, 0.067) |
| Log-CRP | --- | --- | -0.077 **  (-0.107, -0.047) | | -0.063 **  (-0.095, -0.031) |
| BMI | --- | -0.059 **  (-0.089, -0.029) | --- | | -0.035 *  (-0.067, -0.003) |
| Current Smoking | -0.031 *  (-0.061, -0.001) | -0.037 *  (-0.067, -0.007) | -0.023  (-0.053, 0.007) | | -0.028  (-0.058, 0.002) |
| **Model 5**: Effect of current smoking on BMI: β= -0.110 (-0.142, -0.078), p<0.001  **Model 6**: Effect of current smoking on log-CRP: β= 0.100 (0.068, 0.132), p<0.001 | | | | | |

**Table S1**. Standardized regression coefficients of smoking, BMI and CRP on LTL

β=standardized regression coefficients; CI=confidence interval; PA=physical activity; CRP=C-reactive protein; BMI=body mass index; LTL=leukocyte telomere length

* p<0.05 and ** p<0.01 for βs being different from 0
